# The biology and role of CD44 in cancer progression: therapeutic implications

**DOI:** 10.1186/s13045-018-0605-5

**Published:** 2018-05-10

**Authors:** Chen Chen, Shujie Zhao, Anand Karnad, James W. Freeman

**Affiliations:** 1 Department of Cell Systems & Atanomy, University of Texas Health Science Center at San Antonio, San Antonio, TX USA; 2 Department of Medicine, Division of Hematology and Oncology, University of Texas Health Science Center at San Antonio, San Antonio, TX USA; 3 Research and Development, Audie Murphy Veterans Administration Hospital, San Antonio, TX USA; 4 Mays Cancer Center, Experimental and Developmental Therapeutics Program, 7979 Wurzbach Rd, San Antonio, TX 78229 USA

**Keywords:** CD44, Cancer, Signaling transduction, Tumorigenicity, Epithelial to mesenchymal transition (EMT), Cancer therapy

## Abstract

CD44, a non-kinase transmembrane glycoprotein, is overexpressed in several cell types including cancer stem cells and frequently shows alternative spliced variants that are thought to play a role in cancer development and progression. Hyaluronan, the main ligand for CD44, binds to and activates CD44 resulting in activation of cell signaling pathways that induces cell proliferation, increases cell survival, modulates cytoskeletal changes, and enhances cellular motility. The different functional roles of CD44 standard (CD44s) and specific CD44 variant (CD44v) isoforms are not fully understood. CD44v contain additional peptide motifs that can interact with and sequester growth factors and cytokines at the cell surface thereby functioning as coreceptors to facilitate cell signaling. Moreover, CD44v were expressed in metastasized tumors, whereas switching between CD44v and CD44s may play a role in regulating epithelial to mesenchymal transition (EMT) and in the adaptive plasticity of cancer cells. Here, we review current data on the structural and functional properties of CD44, the known roles for CD44 in tumorigencity, the regulation of CD44 expression, and the potential for targeting CD44 for cancer therapy.

## Background

CD44 is a family of non-kinase, single span transmembrane glycoproteins expressed on embryonic stem cells and in various levels on other cell types including connective tissues and bone marrow [1, 2]. CD44 expression is also upregulated in subpopulations of cancer cells and is recognized as a molecular marker for cancer stem cells (CSC) [3]. In humans, CD44 is encoded by 19 exons with 10 of these exons constant in all isoforms. The standard form of CD44 (CD44s) is encoded by the ten constant exons. CD44 variant isoforms (CD44v) is generated by alternative splicing and possess the ten constant exons and any combination of the remaining nine variant exons [4, 5].

The main ligand for CD44 is hyaluronic acid (HA), an abundant component of the extracellular matrix (ECM) that is expressed by stromal and cancer cells [6]. HA binds the CD44 ligand binding domain inducing conformational changes that allow binding of adaptor proteins or cytoskeletal elements to intracellular domains that in turn activate various signaling pathways leading to cell proliferation, adhesion, migration, and invasion [7, 8]. CD44s and various CD44v isoforms have overlapping and distinct functional roles. CD44v isoforms have additional binding motifs that promote the interaction of CD44 with molecules in the microenvironment [9]. CD44v isoforms can act as co-receptors by binding/sequestering growth factors on the cell surface and presenting these to their specific receptors [10]. Cancer cells that undergo an epithelial to mesenchymal transition (EMT) acquire stem cell-like properties and show an increase in CD44 expression [11]. Moreover, cancer cells with an EMT phenotype show increased invasiveness and are more resistant to chemotherapy [12].

The clinicopathological impacts of CD44s and its isoforms in promoting tumorigenesis suggest that CD44 may be a molecular target for cancer therapy (13). In addition, the established role of CD44 in maintaining stemness and the function of cancer stem cells in tumor regeneration following therapy suggests that CD44 may also be an important prognostic marker. Therapeutic strategies that target CD44 or reduce CD44 expression are in various stages of clinical development [13–15]. These strategies include CD44 neutralizing antibodies, tumor delivery of shRNAs, ectodomain mimics, and aptamers [14, 16–18]. Thus, an important area of investigation is to further define the functional roles of CD44 isoforms in cancer and to determine the potential benefits of targeting these CD44 isoforms or their signaling pathways for cancer therapy.

## Structure and function of CD44

### Exon composition and functional domains of CD44

CD44, a non-kinase transmembrane proteoglycan, is a single-chain glycoprotein encoded by one gene located on chromosome 11 in humans and on chromosome 2 in mice. The CD44 gene is comprised of 19 exons in humans and 20 in mice; a homolog of exon 6 or variant 1 is not found in humans [19]. The first five and the last five exons are constant and encode the shortest isoform of CD44 (85–95 kDa) called CD44 standard (CD44s). The middle nine exons can be alternatively spliced and assembled with the ten exons contained in the CD44 standard isoform and are referred to as CD44 variant isoforms (CD44v).

**Fig. 1:**
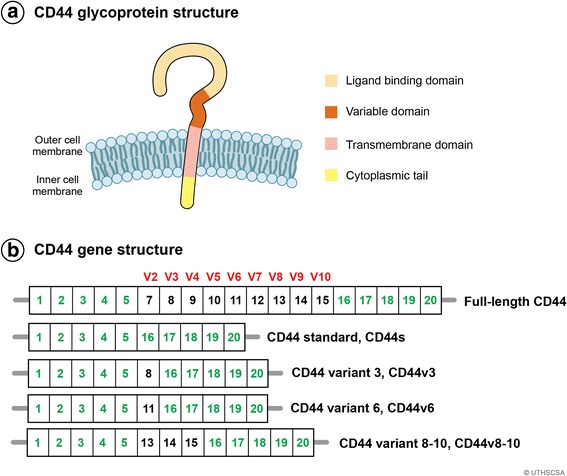
CD44 protein and gene structure. **a** Four domains of CD44 glycoprotein are presented with correponding colors: ligand binding domain, variable domain, transmembrane domain, and cytoplasmic tail. **b** CD44 is encoded by 20 exons in mice but 19 exons in humans. Exon 6 coding for CD44 variant 1 (CD44v1) is lacking in humans. Green color exons are always expressed as a standard form of CD44 (CD44s), and up to nine exon variants can be inserted by alternative splicing. Full-length CD44, CD44s, CD44v3, CD44v6, and CD44v8-10 are shown schematically

CD44v isoforms can be inserted as a single variant exon or combined with other variant exons that code for peptides located in the juxta membrane domain. The encoded CD44 peptide can be further modified by N- and O-linked glycosylation and glycosaminoglycanation by addition of heparin sulfate or chondroitin sulfate [20, 21]. CD44 has extracellular domains, a membrane-proximal region, a transmembrane domain, and a cytoplasmic tail [22]. An illustration of the CD44 gene showing CD44s and CD44v exons and the functional domains and the position of peptides coded by variant exons are illustrated in Fig. 1.

### CD44 ligands and interacting molecules

CD44 binds to several ligands including hyaluronic acid (HA), osteopontin (OPN) [23], chondroitin [24], collagen [25], fibronectin [26], and serglycin/sulfated proteoglycan [27].

HA is the most specific ligand for CD44 activation. All isoforms of CD44 possess a HA-binding domain located in N-terminal region of the extracellular domain (Fig. 1). HA is a major extracellular matrix component expressed by stromal and tumor cells. HA is a linear glycosaminoglycan consisting of repeating disaccharide units of d-glucuronic acid and *N*-acetyl-d-glucosamine and is expressed in various molecular sizes up to 10^7^ kDa. HA binding to CD44 causes conformational changes favoring the binding of adaptor molecules to the intracellular cytoplasmic tail of CD44 leading to cell signaling that enhances cell adhesion, migration, and proliferation [7]. The pathways activated through CD44-HA binding include Ras, MAPK, and PI3K [28]. CD44 binding to HA is cell specific and depends on the activation state of CD44; when HA interacts with CD44, CD44 proteins exist in three scenarios: non-bound to HA, non-bound to HA unless activated by physiological stimuli, or constitutive binding to HA [29]. In breast tumor cells, HA activation of CD44 leads to the expression of multidrug resistance gene (P-glycoprotein) and anti-apoptotic gene Bcl expression, which promotes tumor cell proliferation and survival [30]. Pancreatic cancer cells (MiaPaCa-2) secrete hyaluronidases abundantly and generate readily detectable levels of low molecular weight HA ranging from 10- to 40-mers without any exogenous stimulation. Hyaluronidase-mediated degradation plays a major role in HA oligosaccharide generation in these cells. The tumor-derived HA oligosaccharides enhance CD44 intracellular cleavage and tumor cell motility. Inhibition of CD44-HA interaction results in abrogation of tumor cell motility [31]. Bladder cancer cells transfected with anti-sense to hyaluronic synthases1 (HAS1) showed downregulation in expression of CD44v3, CD44v6, and CD44s both at mRNA and the protein levels indicating that HAS1 regulates the growth of bladder cancer cells by modulating HA synthesis and HA receptor levels [32].

Versican (VCAN) is a chondroitin sulfate proteoglycan that binds to HA leading to structural aggregations of these molecules [33]. VCAN is secreted by the fibroblasts of the tumor-associated stroma and by cancer cells [34]. The carboxyl-terminal domain of VCAN interacts with the β1 integrin of glioma cells, activating focal adhesion kinase (FAK), promoting cell adhesion and preventing apoptosis [35]. Expression of VCAN is increased significantly in breast carcinomas that have malignant-appearing microcalcifications (MAMCs) and mammographic density (MD) as compared to normal breast tissue with MAMCs and MD and normal breast tissue without mammographic findings. MAMCs and MD represent the earliest mammographic findings of non-palpable breast carcinomas. Elevated level of VCAN correlated with higher tumor grade and invasiveness in carcinomas with MD and MAMCs [36]. Artificial expression of versican in different level affects the malignant phenotype of cultured human osteosarcoma cells. Upon binding to leiomyosarcoma cells surface CD44, proliferation of globular domain of VCAN-overexpressing cells were dose dependently inhibited by exogenous HA fragments [37]. TGF-β enhanced the aggressiveness of ovarian cancer cells by upregulating VCAN in cancer-associated fibroblasts (CAF). TGF-β receptor-dependent SMAD signaling transduction regulated VCAN expression in CAFs. Upregulated VCAN promoted the motility and invasion of ovarian cancer cells by upregulating expression of CD44 and receptor of hyaluronic acid-mediated motility (RHAMM) [38]. Thus, interrupting HA-CD44 binding through VCAN may change the proteoglycan enrichment in the tumor lesion to reduce tumor growth or metastasis in cancers.

Osteopontin (OPN) also binds to CD44 inducing cell signaling that mediates tumor progression and metastasis [23]. The expression of OPN and CD44v6 correlated positively in 159 non-small cell lung cancers [39]. Strong correlation of OPN and CD44 expression was detected in 243 gastric cancer patients’ tissues [40]. OPN shared a perivascular expression pattern with CD44 and promoted glioma stem cell-like phenotypes. OPN promotes stem cell-like properties and radiation resistance in adjacent tumor cells via activation of CD44 signaling [41]. CD44-positive colorectal cancer cells co-cultured with macrophages produced higher levels of OPN that facilitated tumorigenicity and clonogenicity. The knock-down of CD44 or inhibitory antibody of CD44 attenuated OPN secretion preventing OPN-activated c-jun-NH_2_-kinase signaling resulting in a decrease in clonogenicity of colorectal cancer cells [42].

*Serglycin* binds to CD44 as its ligand in hematopoietic cells [27]. Glycosaminoglycans composed of chondroitin sulfate are attached to serglycin and may facilitate CD44 binding. The chondroitin sulfate (CS)-type serglycin capable of binding CD44 is secreted by hematopoietic cells including malignant cell lines and normal cells. The expression of serglycin and CD44 core proteins enhanced in breast cancer cells and CS-E subunit attaches to CD44 to promote and regulate breast cancer progression [43]. Therefore, glycosaminoglycan modifying the CD44 binding serglycin differs from one cell type to another [44].

CS is a ligand for CD44 [43] and it protects chronic lymphocytic leukemia (CLL) cells from apoptosis [45]. Combination of CS with gemcitabine strongly inhibited human bladder cancer cells growth [46]. CS-g-poly copolymers can be self-assembled into micelles in water and then used to encapsulate camptothecin. The micellar internalization into lung cancer cells was through CD44 and clathrin dual-mediated endocytosis. The cell killing and apoptosis-inducing effects were better than using drug alone against non-small cell lung cancers in vitro and in vivo [47].

Fibrin is a CD44 ligand in colon carcinoma cells. Platelet-derived growth factor (PDGF) enhanced the adhesion of CD44v-coated beads to immobilized fibrin. PDGF also augmented the binding of CD44v to fibrin by attenuating CD44 sulfation on chondroitin and dermatan sulfate chains. PDGF moderately reduced the sulfation of CD44s and CD44s-fibrin recognition [48].

Several distinct CD44 isoforms co-precipitated with MMP-9 in mouse mammary carcinoma and human melanoma cells and this interaction is believed to help localize MMP-9 to the cell surface. The role of CD44 in promoting tumor invasion may be mediated in part by this binding proteolytically active MMP-9 at the membrane [49]. Rounded-amoeboid melanoma cells secrete higher levels of several matrix metalloproteinases (MMPs) and they degrade collagen I more efficiently than elongated-mesenchymal cells. MMP-9 promoted rounded-amoeboid 3D migration through regulation of actomyosin contractility via CD44 receptor using a non-catalytic mechanism [50].

Fibronectin binds to CD44 indirectly. HA-bound CD44 interacts with fibronectin in the ECM of induced myofibrolasts. Inhibiting HA synthesis promotes fibronectin and collagen deposition [51]. Advanced stage of human colorectal cancer patients show substantially higher levels of fibronectin extra domain A (EDA) in tumor tissue and serum. CD133^+^/CD44^+^ cells expressed significantly elevated EDA receptor than its double negative cells. Silencing EDA in colon cancer SW480 cells reduced spheroid formation and reduced double positive CD133^+^/CD44^+^ cells. Fibronectin EDA may promote tumorigenesis by sustaining the properties of CD133^+^/CD44^+^ colon cancer cells [52]. The predominant CD44 splice variant in prostate cancer-bound fibronectin required HA bound to CD44 [53].

### Functional significance of CD44 isoforms in cancer cells

The roles that CD44 isoforms expression plays in the pathogenesis of cancer are under investigation. Separate isoforms possess overlapping and distinct cellular functions. CD44 can undergo isoform switching in tumor cells as demonstrated by Brown and colleagues [54]. They showed that induction of EMT required a switch in CD44v to CD44s isoform expression. In agreement with isoform switching, our lab recently demonstrated in pancreatic cancer cells that an EMT phenotype was dependent on upregulation of CD44 expression with CD44s being the most prevalent isoform [12]. Other studies indicated that CD44v isoforms were expressed in metastasis in several types of solid tumors [55–57, 15] and were associated with poorer prognosis [58, 59]. This review, although not comprehensive, will discuss some the major studies related to the functional significance of CD44 isoforms.

The different CD44v isoforms encode additional peptides that are inserted in the juxta membrane domain and these cause both conformational changes and provide binding sites for molecules including cytokines and growth factors. It is clear that these different binding motifs influence biologic activity of CD44. Sorting through the functional roles of CD44 isoforms is complicated by the finding that tumor cells may express multiple CD44v isoforms and that these are found in different levels of expression. Many studies related to tumor type, metastasis, and prognosis are correlative and a list of some of these although not comprehensive is shown in Table 1. An overview of these studies is discussed below in relation to different tumor types.

**Table 1:** The significance and function of CD44 isoforms

CD44 isoforms	Biological functions	Cancer types	References
CD44s	Tumor growth, metastasis, low survival rate	Pancreatic cancer	[13]
CD44s	Progression	Breast cancer	[54]
CD44v3	Migration, overexpressed in tumor tissue	Head and neck squamous cell carcinoma	[80]
CD44v3	Proliferation and cisplatin resistance	Head and neck squamous cell carcinoma	[81]
CD44v4, CD44v5	Lung metastasis loci	Pancreatic cancer	[68]
CD44v6	Metastasis; association with liver metastasis	Pancreatic cancer	[60, 61, 67]
CD44v6	Epithelial phenotype cells expression	Prostate cancer	[75]
CD44v6	Tumorigenic and chemoresistance	Prostate cancer	[57]
CD44v6	Migration, metastasis, advanced stage of tumor	Colorectal adenocarcinomas	[15, 63]
CD44v6	Metastasis	Colon cancer	[15]
CD44v6,CD44v9	Correlates with lymph node metastasis, liver metastasis, and TNM stage	Pancreatic cancer	[56]
CD44v9	Lower survival rate, correlates with lymph node/liver metastasis, and TNM stage	Pancreatic cancer	[56]
CD44v9	Associated with worse prognosis, contributed to EMT-mediated invasion and migration	Bladder cancer	[200]
CD44v9	Inhibited assembly of p-cMet, AR, HSP90, P110α/PI3K, and CD44 into lipid raft like structures	Prostate cancer	[76]
CD44v4-10	Promoted adenoma initiation in Apc(Min/+)mice, tumor initiation	Colorectal cancer	[88]
CD44v8-10	Tumor initiation	Gastric cancer	[89]
CD44v8-10	Lung metastasis	Breast cancer	[70]
CD44v (v6-10,v7-10,v8-10)	Correlative study of CD44v expression on transgenic Gan mice	Gastric tumor	[86]
CD44v8-10 overexpression	Enhance chronic phase CML progenitor replacing capacity	Leukemia	[201]
CD44, isoforms not specified	Correlative study of CD44 expression on malignant stage	Prostate cancer	[73]
CD44, isoforms not specified	Increased colony formation, invasion	Prostate cancer	[79]
CD44, isoforms not specified	Poor prognosis, low survival rate, metastasis	Pancreatic cancer	[69]

#### Pancreatic cancer

Human pancreatic cancer tissues express several CD44 isoforms. The most widely studied CD44 variant form in pancreatic cancer is CD44v6. Elevated expression of CD44v6 in pancreatic cancer is reported to play a role in metastasis [60, 61]. Transfection of CD44v6, originally discovered in metastases of a rat pancreatic adenocarcinoma (BSp73ASML), converted non-metastatic carcinoma rat cells into metastatic cells [62]. CD44v6 expression is largely restricted to the advanced stages of clinical development and was more prevalent in metastatic than in non-metastatic adenocarcinomas [63]. BxPC-3 and Panc-1 pancreatic cancer cell lines expressed CD44v3, v4, v5, v6, v7, and CD44s. Interferon (IFN) γ treatment resulted in a slight reduction of the CD44v6 surface expression but not CD44v5 upregulation in Panc-1 cells. Treatment of cancer cells with other growth factors and cytokines (bFGF, EGF, TNFα, and TGF-β1) did not appear to change CD44v expression [64]. Treatment with hepatocyte growth factor (HGF) activated c-Met in two metastatic cancer cell lines BSp73ASML and HT29 and this was inhibited by neutralizing antibodies to CD44v6 [10].

Several correlative studies in human pancreatic cancer suggest that CD44v could be used as markers for clinical prognosis. Immunohistochemically screening revealed different CD44v expression patterns in 24 pancreatic ductal adenocarcinoma (PDAC) patients and human PDAC cell lines: BxPC-3, AsPC-1, MiaPaCa-2, Capan-1, Capan-2, Panc-1, and PaTu II [65]. Fifty percent of cancer tissues showed positive CD44v6 staining and 38% showed positive CD44v2 staining in tissues from 42 separate pancreatic cancer patients but not detectable in normal tissues. CD44v2 was co-expressed with CD44v6 and CD44v2 expression significantly correlated with vessel invasion. Patients with CD44v6-positive primary tumors had a shorter survival time than those with CD44v6-negative tumors [66]. The expression of CD44v6 and integrin-β1 were positively correlated in tumors from 30 pancreatic cancer patients and expression of CD44v6 was significantly associated with liver metastasis. Their expression of CD44s or variants was not associated with the sex, age, and tumor location [67]. A separate study of 101 pancreatic tumor specimens indicated that CD44v6/v9 double positive but CD44s negative tumors had lower overall survival rate and were associated with metastasis. Lower expression of CD44s was detected in metastasis human tissue than non-metastasis tissue [56]. Levels of CD44s were detected significantly higher in 192 human patients’ pancreatic adenocarcinoma tissues than their adjacent non-tumor pancreatic tissues in another study. Patients expressing high levels of CD44s have median survival of 10 months compared with more than 43 months in patients expressing low levels of CD44s.

CD44v5/v6-positive and CD44s-negative cell populations were enriched in metastatic lung foci in orthotopic SCID mice injected with HPAC pancreatic cells [68]. Treatment of mice harboring orthotopically implanted pancreas tumors with a CD44 neutralizing antibody reduces tumor growth, metastasis, and postradiation recurrence [13]. Mice harboring human pancreatic cancer xenografts and injected with CD44v6 peptides showed regression of metastasis. The CD44v6 peptide inhibited c-MET phosphorylation in the primary tumor and HGF and VEGF secretion in L3.6pl cells. The CD44v6 peptide was more efficient than the c-MET inhibitor crizotinib and/or the VEGFR-2 inhibitor pazopanib in reducing xenograft tumor growth and metastasis [14]. Gemcitabine enriched CD44 expression in nude mice implanted with Panc-1 cells which were injected with gemcitabine for 36 days (100 mg/kg/6 days i.p.) [69]. Collectively, these studies suggest that CD44v isoforms are associated with metastases and that chemotherapy may either select for a subpopulation of CD44 expressing cells or may induce the expression of CD44.

#### Breast cancer

CD44 alternative splicing was differentially regulated in a murine model of breast cancer progression, resulting in a shift in expression from CD44v to CD44s during the development of recurrent mesenchymal type of breast tumors [54]. Orthotopic injection of CD44v8-10 expressing breast cancer cells in mice resulted in an increase in lung metastasis [70]. The functional role of CD44 on cancer cell metabolism was evaluated by knock-down of CD44 via retroviral delivery of shRNA against CD44 in human breast cancer cells. Silencing of CD44 decreased the glucose uptake of cancer cells, ATP production, and lactate production [71]. Gain- and loss-of-function analysis for CD44v6 has been studied in endocrine-sensitive breast cancer MCF-7 cells. Overexpression of CD44v6 activated epidermal growth factor receptor (EGFR) signaling, enhanced their endogenous invasive capacity, and attenuated their response to endocrine treatment. There was an association between suppression of CD44v6 and a reduction in their invasive capacity in endocrine-resistant breast cancer cells [72].

#### Prostate cancer

One hundred forty-eight prostate tissues composed of prostate cancer (PCa), high-grade prostatic intraepithelial neoplasia (HGPIN), and benign prostate hyperplasia (BPH) were immunostained for CD44 and CD133. A higher level of CD44 expression was observed in 42% of PCa, 57% of HGPIN, and 42% BPH tissues, suggesting that CD44 expression was not correlated to malignant stage of prostate cancer [73]. The roles of CD44 isoforms have also been investigated in prostate cancer. The expression levels of CD44s and all its 9 variants were analyzed in 94 human surgical specimens, whereas the control group consisted of 14 specimens from patients with benign prostatic hyperplasia. In localized prostate cancer, CD44s was underexpressed and all the other isoforms were overexpressed. A higher expression of CD44v2 independently correlated with a better recurrence-free survival rate [74]. RNAseq was used to determine the differential expression of membrane proteins on PC3 cells that possessed epithelial or mesenchymal phenotypes. PC3-epithelial cells had a higher level of CD44v6 as confirmed by flow cytometry and Western blot analysis [75].

The functional role of CD44v6 in prostate cancer cells was assessed using a small interfering RNA (siRNA) approach. Knock-down of CD44v6 caused a loss of EMT markers and reduced tumorigenic potential, tumor sphere formation, and enhanced chemo/radiosensitivity [57]. A separate study showed that silencing CD44v9 using short hairpin RNA inhibited assembly of p-Met, AR, HSP90, P110α/PI3K, and CD44 into lipid raft-like structures and reversed the assembly of these components in the complex in prostate cancer. HGF-induced activation of HGF-receptor/c-Met and stimulated hyaluronan/CD44v9 signaling transduction. This, in turn, stabilized the androgen receptor functions in prostate cancer cells [76].

The roles of miRNAs on CD44 expression have been examined in PCa models. miR-34a, miR-106a, miR-141, and let-7b were downregulated on stem/progenitor cells which expressed CD44. miR-301 and miR-452 were overexpressed on CD44-positive cells [77]. CD44 is predicted as a direct target of miR-199a-3p using target prediction program and this was partially confirmed using a luciferase reporter assay in PCa cells [78]. miR-383 expression repressed CD44 mRNA and protein expression in DU145/PC3/LNCaP cells [79]. These studies indicate that miRNAs play a role in regulating CD44 expression in PCa.

#### Head and neck squamous cell carcinoma

CD44v3 levels were found elevated in head and neck squamous cell carcinoma (HNSCC) cell lines and these higher level of CD44v3 caused a significant increase in cell migration [80]. Transfection of the CD44v3 isoform into a non-expressing HNSCC cell line resulted in increased tumor cells migration. Treatment of anti-CD44v3 antibody in those cells resulted in a decrease in vitro proliferation and cisplatin resistance [81]. Injection of 50 cells of CD44v3^High^, aldehyde dehydrogenase-1 (ALDH1) ^High^ cell populations but not CD44v3^Low^, ALDH^Low^ cells or unsorted cells from tumor-derived human HNSCC formed tumors with high efficiency in NOD/SCID mice. HA treatment of CD44v3^High^, ALDH^High^ cells stimulated a higher amount of Oct4-Sox2-Nanog accumulation [82]. Our lab showed that a flow sorted pancreatic cancer cell population (CFPAC1-CD44^Low^ cells) that expressed low levels of CD44s but with expression of multiple CD44 variant isoforms also expressed higher levels of ALDH. On the other hand, cells that expressed high levels of CD44 standard isoform have relative low level of ALDH (unpublished data). This finding suggests that CD44v expression correlated with ALDH levels. Delta Np-63 directly regulates CD44 expression which potentiated EGFR activation and the expression of ABCC1 multidrug transporter gene which contributed to tumor cell proliferation and chemoresistance in HNSCC [83]. ALDH was a widely accepted property of CSC suggesting that CD44v may relate to stemness properties than CD44s but further functional studies need to be done in context with different cancer stages and cancer types.

#### Gastrointestinal cancer

Poor prognosis in colorectal cancer is correlated with CD44v6 expression [58, 59]. CD44v6 and CD44v10 mRNA were detected in colorectal adenocarcinoma patients [84]. Colorectal CSC expressed CD44v6 which was required for their migration and generation of metastatic tumors. Cytokines such as HGF, OPN, and stromal-derived factor 1α (SDF-1) secreted in the tumor microenvironment increased CD44v6 expression in CSC and activated the Wnt/β-catenin pathway which promoted migration and metastasis [15].

CD44v9 overexpression was found in gastric adenocarcinoma [85]. CD44v (v6-10, v7-10, v8-10) was predominantly expressed in the gastric tumors of transgenic gastric carcinogensis mice model [86]. Ishimoto et al. showed that the direct interaction of CD44v8-10 with a glutamate-cystine transporter can protect gastrointestinal cancer cells against reactive oxygen species [87]. Knock-in mice expressing CD44v4-10 promoted adenoma initiation in Apc (Min/+) mice but not in CD44s knock-in mice [88]. CD44v8-10 expression also confers to gastric tumor initiation. Gain- and loss-of-function analysis of CD44v8-10 revealed that injection of the low numbers of CD44v8-10-depleted cells (200 cells) did not form tumors compared with CD44v8-10-expressing cells. CD44v8-10 but not CD44s expression rescued the tumor-initiation potential of gastric cells in which total CD44 was depleted [89]. Overall, certain CD44 variants display different functional roles than CD44s in tumor growth. Therefore, the importance of each CD44v and how it relates to tumor progression is of interest. It is also likely that different CD44 variants will have overlapping as well as distinct functions. Cells overexpressing CD44v6 that were treated with chemotherapy drugs showed a higher viability and an increased clonogenicity in colon cancer cells compared to the control cells that lacked CD44v6 expression. Moreover, overexpression of CD44v6 resulted in enhanced autophagy flux, EMT, and phosphorylation of AKT and ERKs in the presence of chemotherapy drug [28].

CD44v6 expression correlates with clinicopathological characteristics and prognosis in gastrointestinal patients. STAT3 inhibitor AG490 decreased CD44v6 expression which is regulated by IL-6/STAT3 signaling pathway in cancer cells [90]. Metformin delayed a tumor growth in a patient-derived-xenograft mouse model and decreased the self-renewal ability of the gastric CSC which highly express CD44 and Sox2 [91]. Both studies provide the therapeutic perspectives to target CD44 expression in patients.

## Regulation of CD44

**Fig. 2:**
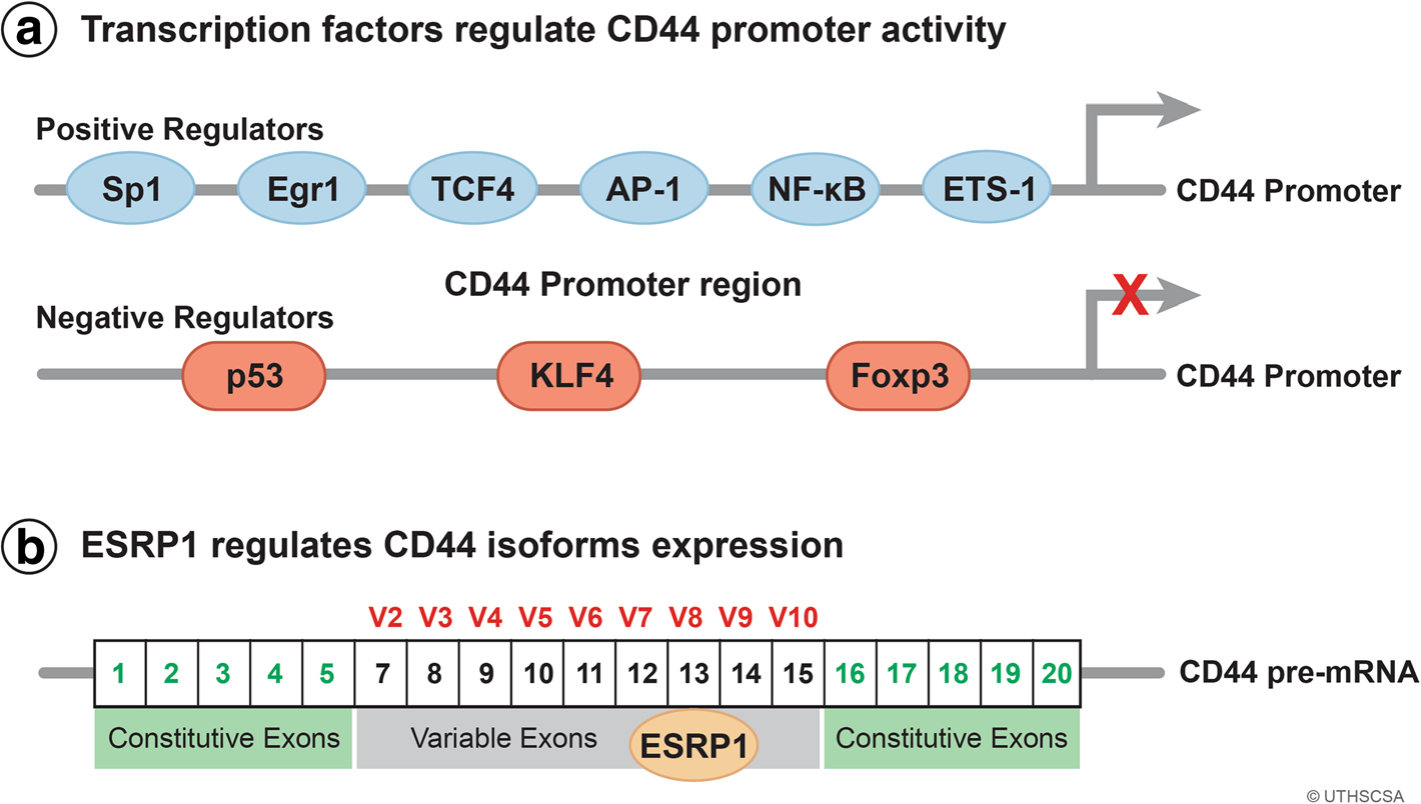
Molecules regulate promoter activity and expression. **a** Several transcription factors including positive or negative regulators bind to the CD44 promoter region and regulate its activity. **b** Epithelial splicing regulatory protein 1 (ESRP1) is required for alternative splicing which generates different CD44 variant isoforms

Because of the contribution of CD44 to cancer cell function; it is important to understand how CD44 expression is regulated. Emerging data show that specific signaling networks can induce CD44 expression. Specific transcriptional repressors and activators have been identified to regulate CD44 promoter activity. Moreover, epigenetic mechanisms and miRNAs are implicated in regulating CD44 expression. Understanding the mechanisms of regulating CD44 expression may provide molecular targets that could be used to modulate CD44 expression as a means of mitigating CD44 oncogenic function. Several representative regulatory mechanisms are shown below in Fig. 2.

### Transcription factors

The structural analysis of CD44 upstream regulatory region in neuroblastoma cell lines revealed it lacked TATA and CCAAT consensus sequences. Three copies of the hexanucleotide sequence GGGCGG was found upstream of CD44 transcription initiation site. These sequences typically bind the transcription factor Sp1 that is known to drive transcription of promoters that lack TATA boxes [92].

The regulatory regions of the CD44 promoter were determined using serial deletion mutants of the CD44 promoter linked to a luciferase reporter [93]. Several transcriptional factors including Sp1, Egr1, P53, NF-κB, and ETS-1 bound to the CD44 promoter region inducing promoter activity and expression [94–96, 92, 97].

An Egr-1 binding motif was identified 301 base pairs upstream of the human CD44 transcription start site. Co-transfection of a CD44 promoter-chloramphenicol acetyltransferase reporter construct with an Egr-1 expression vector resulted in increased transcriptional activity [96]. Binding assays identified components of AP-1 and NF-κB binding to the conserved region located upstream of the CD44 promoter. Chromatin immunoprecipitation (ChIP) results confirmed that AP-1 and NF-κB p50, p65 bound strongly with above conservative region in breast cancer cells [98]. A separate study in triple-negative breast cancer cells showed that NF-κB and AP-1 were key transacting factors that interact with this conserved region. A pharmalogical inhibitor of NF-κB also reduced CD44 expression and consequently, cancer cell proliferation and invasiveness were decreased [95].

An ETS-1 (v-ets erythroblastosis virus E26 oncogene homolog 1) binding site was also identified on the CD44 promoter. Sphingosine-1-phosphate (S1P) treatment stimulated the binding of ETS-1 to the CD44 promoter region. Moreover, S1P induced the expression and nuclear translocation of ETS-1. Knock-down of ETS-1 inhibited the S1P-induced CD44 expression and human lung cancer cell migration [94].

Ataxia telangiectasia group D complementing gene (ATDC) upregulated CD44 expression in mouse and human PanIN lesions (pancreatic intraepithelial neoplasia) via activating β-catenin signaling. ChIP assays along with transcription element search system revealed TCF4 binds to CD44 promoter region, 1500 base pairs upstream of the CD44 transcription initiation site. β-catenin/TCF signaling induced CD44 expression, giving rise to EMT characterized by expressing Zeb1 and Snail1 in PanIN [97].

Foxp3 and CD44 protein levels were inversely correlated in several breast cancer cell lines. Overexpression of Foxp3 in two breast cancer cell lines downregulated both mRNA and protein expression levels of CD44. Transient knock-down of Foxp3 from breast cancer cell lines upregulated both mRNA and protein level of CD44, indicating that Foxp3 is a negative regulator of CD44. The effect of Foxp3 on regulation of the CD44 promoter activity in breast cancer cells was assessed by ChIP and EMSA analysis. One potential Foxp3 binding motif was identified at 810 base pairs upstream of the CD44 transcription start site. Whether Foxp3 directly binds to CD44 promoter acting as a repressor of CD44 transcription needs further confirmation [99].

P53 inhibits expression of the CD44 cell-surface molecule via binding to a noncanonical p53-binding sequence in the CD44 promoter. CD44 mRNAs were upregulated four- to fivefold and CD44 protein increased fourfold in BPEC-T cells in which p53 function was inhibited. Direct influence of p53 on the promoter of the CD44 gene was further confirmed using a luciferase reporter assay [93].

KLF4 is a negative regulator of CD44 promoter activity. Genetic ablation of KLF4 in pancreatic cancer cells isolated from KLF4 ^flox/flox^ mice significantly increase CD44 expression. KLF4 acts as a CD44 repressor by binding to CD44 promoter region to negatively regulate the transcription of CD44 and its variants [100]. KLF4 is a dominant regulator of the epithelial/mesenchymal status in human breast epithelial cells. KLF4 overexpression induced a switch from a mesenchymal to epithelial state in breast cancer cells. In this context, the induction of mesenchymal phenotype in vitro and metastasis in vivo in breast cancer cells requires downregulation of KLF4 [101]. Targeted activation of KLF4 for therapeutic intervention of tumors has been approved for a clinical trial [102].

A study showed that knock-down of the stem cell factor Sal-like protein 4 (SALL4) decreased CD44 expression. The results of luciferase-promoter assay and a ChIP assay demonstrated that SALL4 bound to CD44 promoter region and transcriptionally activated CD44. CD44 overexpression antagonized SALL4 knock-down-mediated inhibition of gastric cancer cell proliferation, migration, and invasion in vitro and gastric cancer growth in vivo. Therefore, SALL4 likely promotes gastric cancer progression, at least in part, by directly activating CD44 expression [103].

In addition, cytokines and growth factors may stimulate signaling cascades critical for activation of transcription factors that drive CD44 promoter activity. CD44 transcriptional activity was induced by co-transfecting c-Ha-ras expression constructs and CD44 promoter reporter constructs and this required AP-1 binding [104].

**Fig. 3:**
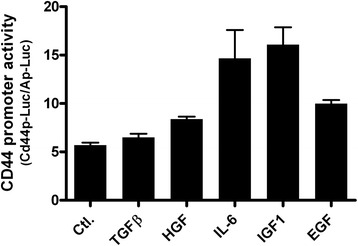
CD44 promoter activity comparison among different treatment of cytokines groups. TGFβ, HGF, IL-6, IGF1, and EGF treated on CFPAC-1 CD44 low cells. *TGFβ* transforming growth factor β, *HGF* hepatocyte growth factor, *IL*-*6* interleukin-6, *IGF1* insulin-like growth factor 1, *EGF* epidermal growth factor

Cytokines and growth factor stimulation may also promote CD44 transcription presumably by activating transcriptional factors. Exogenous IL-1β caused a dose- and time-dependent induction of CD44 expression in rat aortic smooth muscle cells and increased the rate of CD44 gene transcription within 8 h of stimulation [105]. Our lab tested the effects of several growth factors/cytokines on CD44 promoter activity and showed that both IL-6 and IGF-1 can induce CD44 promoter activity in pancreatic cancer cells in Fig. 3.

CD44 expression is also linked to β-catenin and AKT pathways in breast cancer and cervical cancer cells. Inhibition of both β-catenin expression and AKT pathway suppresses expression of CD44 greater than inhibiting either pathway alone suggesting that the activation of these two pathways was critical for the maintenance of CD44 expression [106].

Rac1 activity is increased in gastric adenocarcinoma spheroid which expressed high level of CD44. Rac1 knock-down or its inhibitor decreased CD44 expression and spheroid formation. This suggested that Rac1 may be upstream regulator of CD44 expression [107]. Aggressive oral cancer cell lines from a murine-oral-cancer model (MOC2, 7, 10) express CD44 and phosphorylated ERK1/2. Intracellular phosphor-ERK1/2 staining coincided with CD44 in MOC2. Blockage of ERK1/2 by U0126 decreased CD44 surface expression. When MOC1 cells were co-transfected with CD44 full-length promoter reporter and a tamoxifen-inducible MEK1/R4F construct, tamoxifen induces CD44 promoter activity [108]. These studies suggest that Rac1 and ERK1/2 signaling regulate CD44 expression and blocking CD44 upstream signal may provide therapeutic strategy.

### Epigenetic regulation

Hypermethylation of the 5′ CpG island of the CD44 gene was observed in 31 of 40 primary prostate cancer specimens and was associated with CD44 transcriptional inactivation [109]. But whether CD44 acts as suppressor gene in human prostate cancer in different stage is still under debate. Methylation of the CpG islands in the promoter region of CD44 might account for CD44 downregulation. Pyrosequencing analysis suggests DNA methylation changes in a variety of genes regions including CD44 in triple negative breast cancer [110].

Treatment of different tumor cells, DU145, LNCaP, and MCF-7 cells, with the demethylation agent 5-aza-CdR changed the histone code and methyl cytosine phosphate guanine (CpG)-binding domain protein (MBD) profile at the promoter regions of CD44, Cyclin D2, GLIPR1, and PTEN. This change was associated with upregulation of mRNA expression of these genes [111]. In retinoid-maturation-resistant NB4-LR1 sub clone, CD44 gene was silenced. The molecular defect involved DNA methylation of CpG island and underacetylation of histone H3 on the CD44 promoter [112].

### Regulation of CD44 by miRNAs

MicroRNAs (miRs) are a conserved class of noncoding RNAs that regulate a variety of biological processes, including cancer progression. They regulate gene expression by binding to mRNA leading to mRNA degradation or inhibition of translation. Several miRNAs are reported to regulate CD44 expression [113, 114]. Pancreatic cancer MiaPaCa2 and BxPC3 cells express low levels of both primary and mature miR-34a, b, c but high levels of the miR-34 target genes BCL2 and Notch1, and different levels of Notch2–4. Only 1–2% of MiaPaCa2 cells are CD44^+^/CD133^+^ double-positive. CD44^+^/CD133^+^ double-positive cells grow typical tumor spheres. Those cells had a high level of Bcl-2 expression but loss of miR-34a/b/c compared with CD44^−^/CD133^−^ cells or the unsorted (total) cells. Restoration of miR-34a significantly decreased the CD44^+^/CD133^+^ cells. miR-34a-induced reduction of the CD44^+^/CD133^+^ cells were accompanied by reduced number and size of tumor sphere formation. Similar results were confirmed in BxPC3 cells, where lentiviral miR-34a restoration significantly inhibited the clonogenic growth and tumor spheres. Taken together, CD44 is a downstream target of miR-34a in pancreatic cancer [115]. In prostate cancer, infection of lenti-miR34a in CD44-positive Du145 cells completely blocked tumor development. On the other hand, applying anti-miR34a or miR-34a antagomir to CD44-negative cells promoted tumor growth [113]. Functional studies in both pancreatic and prostate cancer identified that CD44 was a direct and functional target of miR-34a. Knock-down of CD44 photocopied miR-34a overexpression by inhibiting prostate cancer regeneration and metastasis [113].

Constructs containing the putative miR-328 target site or a mutated sequence of the 3′-UTR of CD44 clone downstream of a luciferase reporter gene were used to see whether miR-328 directly targeted the 3′-UTR of CD44. HCT116 cells transfected with a miR-328 mimic significantly suppressed luciferase activity from the reporter containing the wild-type CD44 3′-UTR compared with the control vectors [116]. In addition, osteoblast and osteosarcoma cell lines KHOS and U-2OS exhibit significantly higher expression of CD44. Similar effect was seen in human tumor tissues. Higher expression of CD44 was found in both patients with shorter survival and patients who exhibited unfavorable response to chemotherapy before surgical resection. miR-199a-3p repressed CD44 expression in both a dose-dependent and time-dependent manner. Transfection of miR-199a-3p significantly restored the sensitivity to chemotherapeutic drug doxorubicin in U-2OS cells suggesting that CD44 was a downstream target of miR-199a-3p in osteosarcoma [114]. A luciferase reporter assay demonstrated that miR-143 directly targeted the 3′-UTR of CD44 in breast cancer cells. miR-143 inhibited breast cancer cells invasion and reduced tumor growth in a mouse model of breast cancer [117].

### Post-transcriptional regulation

CD44 isoforms switching was reported to contribute to cancer metastasis associated with variant isoforms [15, 68, 70, 54]. Epithelial splicing regulatory protein (ESRP) 1 was essential for promoting exon inclusion of CD44 isoforms and was required for expression of CD44v isoforms [118]. ESRP1 regulates CD44 posttranscriptionally by exerting a differential effect on protein translation via 5′-UTRs of mRNAs [119]. The decline in ESRP1 expression coincided with the switch in expression from CD44v to CD44s. Knocking down ESRP1 in CD44v-expressing cells resulted in an isoform switch to CD44s, leading to suppression of lung colonization [70].

## Molecular targets of CD44 activation

**Fig. 4:**
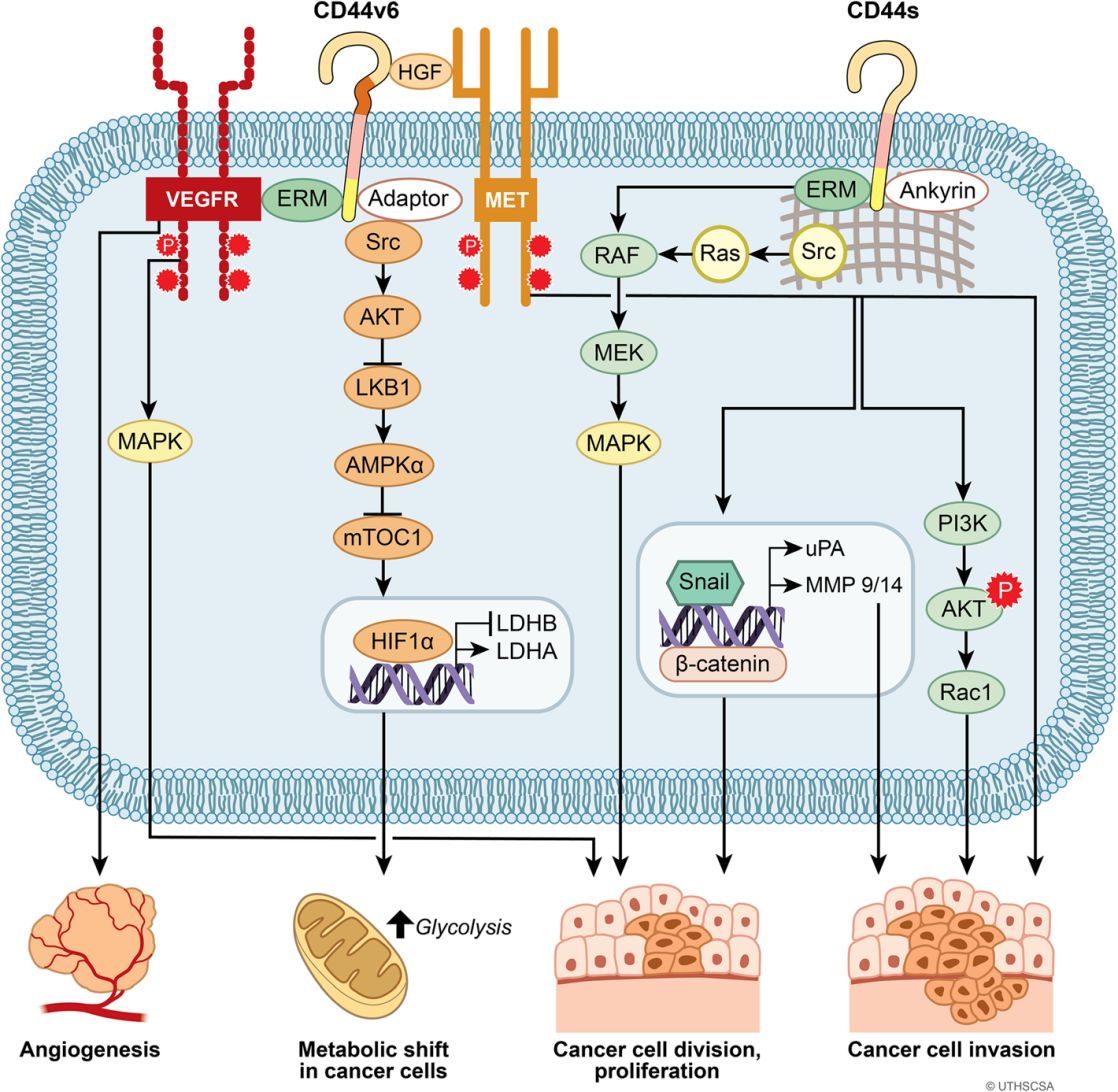
CD44-mediated downstream signaling pathways. CD44s and CD44v6 are shown as representative CD44 isoforms that mediate specific downstream signaling pathways. CD44v6 can recruite ezrin/radixin/moesin (ERM) proteins that promote cytosketal changes and that may interact with VEGFR contributing angiogenesis, cancer cell division, and proliferation. CD44v can act as a coreceptor for the receptor tyrosine kinase c-Met to promote cancer cell invasion. CD44s can change cytoskeleton structure mediating Snail/β-catenin translocation to the nucleus promoting transcrition of the matrix metalloproteinases (MMP) family or urokinase-type plasminogen activator (uPA) expression causing an increase in cancer cell invasion. CD44s can also enhance activation of the PI3K/AKT pathway contributing to cancer cell invasion and proliferation. CD44 modulates Src/MAPK signaling pathway leading to cancer cell division and proliferation. CD44 also causes Hypoxia-inducible factor 1α (HIF1α) binding to nuclear DNA to increase glycolysis, in turn rendering a metabolic shift in cancer cells

CD44 mediates multiple signaling pathways including protein kinases, cytoskeletal changes, intracellular pathways, proteinases, and transcriptional factors to contribute cancer cell division, proliferation, invasion, and angiogenesis as well as metabolic shift (Fig. 4).

### Protein kinases

CD44 activation was attributed to the activation of protein kinase pathways. The activation of these pathways played a role in promoting drug resistance, tumor invasiveness, and other oncogenic properties. Although CD44 lacks kinase activity, there are several mechanisms by which CD44 can activate protein kinases.

CD44 acts as a co-receptor by binding and sequestering growth factors at the cell surface and helps to stabilize tyrosine kinase receptor (RTK) complexes [120]. For example, CD44v6 contains an extracellular peptide domain that binds to HGF, the ligand for c-Met. This binding motif is coded by exon v6 of the CD44 gene. The sequestered HGF bound to CD44v6 is presented to and activates c-Met [10]. Neutralizing antibodies to CD44v6 inhibit c-Met activation. CD44v6 and VEGFR-2 form a constitutive complex that can be detected by co-IP. A CD44v6-specific peptide as well as a CD44v6 neutralizing antibody blocked c-Met activation on endothelial cells and also abrogated VEGF-induced VEGFR-2 activation and downstream ERK activation [121]. Silencing of CD44v6 decreased phosphorylated ErbB2 in colon cancer cells suggesting that CD44v6 may play a role in the activation of this oncogenic pathway [122].

Activation of epidermal growth factor receptor (EGFR) promoted the CSC phenotype with CD44^+^/CD24-expression, associated with invasiveness in triple-negative breast cancers. ERK1/2 activation was necessary for EGFR-induced expansion of CD44^+^/CD24^−^ cell populations. Sustained activation of ERK1/2 by overexpression of constitutively active MEK1 was sufficient to expand CD44^+^/CD24^−^ cell populations in which EGFR activity was blocked by either Erlotinib, an EGFR kinase inhibitor, or BB-94, a metalloprotease inhibitor, suggested that metalloprotease-dependent activation of EGFR modulated CD44^+^/CD24^−^ in triple-negative breast cancer cells through the MEK/ERK pathway [123]. Our lab also observed higher ERK1/2 activation in CD44-positive pancreatic cancer cells than in its counterpart of CD44 knock-down clones (unpublished data). CD44 also contributes to the activation of Kras-mediated signaling through MAPK pathway in lung cancer cell line models. In these cell line models, CD44 promotes tumor cell proliferation and cell survival. Moreover, deletion of CD44 conferred a survival advantage to mice expressing oncogenic Kras^G12D^ in the lung. CD44 knock-down inhibited ERK 1/2 activation [124]. These findings suggest that ERK might be a CD44 downstream target. Transient knock-down of CD44 in human colon cancer cells resulted in upregulation of AKT, whereas overexpression of variant isoforms of CD44 such as v3-10 or v8-10 caused deactivation of AKT. Inhibition of phosphorylation of AKT downregulated cofillin expression and transient knock-down of CD44 decreased cofillin expression [125]. The expression and activity of cell migration-related proteins, including c-Src, paxillin, and FAK, were decreased by CD44 silencing [126]. CD44 may contribute to both the expression and the activation of non-receptor kinases such as the Src family, c-Abl, and Jak that control eukaryotic cell growth and differentiation. Silencing CD44 suppressed both mRNA and protein levels of c-Src and its downstream MAPK pathway. Retroviral-mediated CD44 knock-down decreased cell proliferation, migration, and invasion of breast cancer cells [126]. HA activation of CD44 caused an activation of c-Src in breast cancer cells [127]. CD44-mediated cell spreading and induced tyrosine phosphorylation were prevented by the Src kinase inhibitor, PP2 in murine BW5147 T lymphoma cells [128].

CD44 knock-down decreased the level of FGFR2 expression and inhibited gastric tumor growth, whereas CD44 activation led to upregulation in FGFR2 expression. In turn, FGFR2 kinase inhibitor or its neutralizing antibody decreased the numbers of CD44-positive cells. Conversely, FGFR2 overexpression increased CD44 expression and accelerated tumor growth in mice indicating the positive feedback mechanism in the regulation of CD44 and FGFR2 expression [129]. Silencing of CD44 in human breast cancer cell lines suppressed activation of a signaling axis involving c-Src/AKT/LKB1/AMPKɑ/HIF-1ɑ [71]. Collectively, these studies suggest that CD44 may act indirectly in regulating the expression of protein kinases and their ligands.

### Cytoskeletal changes mediated by activated CD44

Conformational changes of CD44 as a result of ligand binding provide active sites for Ezrin-Radixin-Moesin (ERM) and other adaptor proteins that bind the cytoplasmic tail of CD44. ERM activation as a result of CD44 interaction initiates cross-talk with multiple pathways. For example, activation of c-Met requires sustained activation of CD44-dependent phosphorylated of ERM and Src signaling through the Ras-MAPK and the PI3K-AKT pathway [126, 130]. The binding of ERMs and adaptor proteins to the cytoplasmic portion of CD44 initiates changes in tumor cell cytoskeletal architecture and in cell signaling. CD44 extracellular domain was required for c-Met activation, whereas its cytoplasmic domain recruits ERM proteins that bind the cytoskeleton and promotes Ras activation [131]. In non-migrating cells, CD44 mainly localizes in rafts and Ezrin in nonraft compartments. During migration, CD44 interaction with threonine-phosphorylated ERM proteins in the nonraft compartments was increased. Pharmacological raft disruption increases colocalization of CD44-ezrin as determined by co-IP and colocalization studies [132].

### Intracellular pathways, proteinases, and transcriptional factors

CD44 expression and isoform type is thought to modulate the activities of multiple cellular signaling components. Some examples of how CD44 influences the activities of cell signaling were listed and briefly discussed below.

#### Hippo signaling

Glioblastoma cells depleted of CD44 respond to oxidative stress with robust and sustained phosphorylation/activation of MST1/2 and Lats1/2, phosphorylation/inactivation of YAP, and reduced expression of cIAP1/2. CD44 acts as upstream of the mammalian Hippo signaling pathway (merlin-MST1/2-Lats1/2-YAP-cIAP1/2) and suggests a functional role of CD44 in attenuating tumor cell responses to stress and stress-induced apoptosis [133].

#### β-catenin

CD44 signaling plays a role in regulating the expression and activity of β-catenin. Decreased levels of both mRNA and protein levels of β-catenin were detected in CLL cells where CD44 was knocked down using shRNA. CD44 downregulation decreased the expression of β-catenin, increased the expression of phosphorylated β-catenin, and inhibited cell proliferation in vivo [134].

#### TGF-β

The TGFβ2 is identified as a CD44 downstream target that promotes EMT and breast cancer invasion. TGF-β2 was upregulated when CD44 was induced; inhibition of CD44 gene by RNAi decreased TGF-β2 expression upon HA-stimulation and subsequently inhibited cell invasion in vitro [135]. Activation of the TGF-β2 pathway in human cancer cell lines and in mouse models can induce EMT and promote tumor invasion in culture and to metastasize to distant organ [136–138].

#### Emmprin (CD147)

The immunoglobulin superfamily glycoprotein emmprin (CD147) is a multifunctional glycoprotein that can modify the tumor microenvironment by activating proteinases, inducing angiogenic factors in tumor and stromal cells. CD147 was originally identified as a factor on the surface of tumor cells that induces MMP production in fibroblasts [139]. CD147 can also form complexes with CD44 and EGFR in lipid raft-like membrane domains and induce hyaluronan-CD44-dependent EGFR-Ras-ERK signaling that promoted invadopodia activity and invasiveness in breast epithelial cells [140]. CD147 co-localizes with CD44 at the membrane of MDA-MB231 cells and MCF-7 cells. Perturbation of hyaluronan-CD44 interaction or downregulation of CD147 suppressed lactate efflux in the plasma membrane. Interactions among hyaluronan, CD44, and CD147 also contributed to the glycolytic phenotype of breast cancer cells [141].

#### Matrix metalloproteinases

Transcriptional factor Snail1 is a CD44 downstream target that regulates MMP14 expression and in turn MMP14 is required for pancreatic cancer invasion [142]. Knock-down of CD44 decreased mRNA expression of MMP9, MMP14, and urokinase plasminogen activator (uPA) and inhibited cancer cell invasion in the basal-like human breast cancer cells [143]. mRNA levels of MMP2 and MMP9 were elevated in CD44 positive breast cancer cells compared to CD44-negative cells. Transient knock-down of Smoothened (Smo), a critical component of the hedgehog signaling pathway, inhibited the expression of MMP2 and MMP9 in CD44-positive cancer cells. A correlation of MMP2/9 and CD44 expression was established in cancer cell line models suggesting that inhibiting the CD44-MMP axis might provide therapeutic targets for suppressing metastasis [144]. Thus, these studies support a role of CD44 in regulating the activities of matrix metalloproteinases (MMPs) linked to tumor cell invasion.

#### STAT3

Several studies in different cancer types link CD44 with activation of signal transducer and activator of transcription 3 (STAT3). CD44 forms a complex with STAT3. Gemini, a vitamin D analog decreased CD44 protein levels and inhibited activation of STAT3 signaling by reducing the amounts of CD44v and CD44s proteins interacting with STAT3 [143]. Combination of the natural compounds curcumin and epigallocatechin gallate (EGCG) reduced CD44-positive breast CSC by downregulating the level of phosphorylated STAT3. The CD44-STAT3 axis was reported as a potential target for human breast cancer [145]. CD44 was also shown to regulate gastric stem cell proliferation through activation of STAT3 [146].

#### CD44 and microenvironment

Tumor microenvironment plays an active role in promoting tumor progression since the growth factor or ligand secreted by CAF may interact with CD44 causing malignant phenotype or triggering tumor promoting downstream signaling. Serglycin, secreted by CAF, promotes NSCLC cell migration and invasion as well as colonization in the lung and liver in a CD44-dependent manner. It promotes EMT by enhancing vimentin expression via CD44/NF-κB/claudin-1 (CLDN1) axis [147]. CD44-deficient CAFs do not sustain CSC suggesting that CD44 expressed on CAFs serves as a functional molecule that contributes to the maintenance of cancer stem cell populations in the tumor microenvironment [148]. Cell survival and drug resistance analysis showed CD44-positive CAFs promoted breast cancer cell survival and paclitaxel resistance and inhibited paclitaxel-induced apoptosis [149].

## Roles of CD44 in tumorigenicity

CD44 activating and modulating a number of cell signaling networks that play an important role in mediating tumorigenic properties of tumor cells leading to tumor progression, metastasis and chemoresistance.

### EMT, invasion, and metastasis

CD44 is thought to play a role in the adaptive plasticity of cancer cells [12]. Adaptive plasticity of cancer cells is a term used to explain how a phenotypic change in response to the microenvironment that provides cancer cells a selective growth and survival capabilities. EMT is a form of adaptive plasticity and it is exemplified by a change from an epithelial to a mesenchymal-like phenotype and these cells show increase in motility, are more invasive, and are generally more resistant to apoptosis [11]. However, the development of metastatic lesions likely requires these cells to re-establish their epithelial state through the reverse mesenchymal to epithelial transition (MET), which is believed to favor tumor engraftment and growth of cancer cells at metastatic sites [141]. Major changes during EMT accompanied with loss of epithelial adhesion receptors like E-cadherin, occludin, claudins, and α-catenin as well as disassociation of β-catenin from adherents junctions. At the same time, there is upregulation of expression of molecules associated with a mesenchymal phenotype including vimentin, fibronectin, N-cadherin, and α-smooth muscle actin [93, 150]. This results in a change in cell shape and apico-basal polarity. The functional role of CD44 in EMT has been studied in several cancers. In colon cancer cells, a mesenchymal phenotype was associated with an increase in CD44 and knock-down of CD44 showed a decrease in EMT phenotype. Overexpression of CD44 downregulated E-cadherin expression, upregulated N-cadherin, α-actin, vimentin, fibronectin, and inhibited the formation of the membrane-associated E-cadherin-β-catenin complex, which resulted in cell invasion and migration. CD44 knock-down cells show a marked decreased migration and invasion, CD44 overexpressing cells showed significantly increased cell migratory and invasive capacity [151]. Expression of twist in breast cancer MCF7 and cervical cancer Hela cells induced EMT and the expansion of the cells that were CD44^High^CD24^Low^, which is associated with cancer stem cell properties [106]. CD44 isoform switching from expressing CD44v to CD44s is essential for EMT and breast cancer progression [54]. Breast cancer cells with ectopic expression of ATF3 displayed more elongated and scattered phenotype and the percentage of CD24^Low^ CD44^High^ cell populations was increased [152]. These studies suggest CD44 isoforms expression positively correlates with mesenchymal phenotype and metastasis in variety cancers.

CD44 alternative splicing was differentially regulated during EMT, resulting in a switch in expression from CD44v to CD44s in human immortalized epithelial mammary cells and in a murine model of breast cancer progression [54]. CD44s isoform promotes EMT and its knock-down inhibits EMT. CD44s re-expression fully rescued the impaired EMT phenotype in the CD44-knock-down cells with decreased expression of epithelial markers E-cadherin and occludin and increased expression of mesenchymal markers N-cadherin and vimentin. ESRP1 regulates CD44 alternative splicing to promote exon inclusion of CD44 isoforms and is therefore required to regulate CD44v expression [118]. ESRP1 regulates CD44 posttranscriptionally by exerting a differential effect on protein translation via 5′-UTRs of mRNAs [119]. ESRP1 is expressed highly in cells with an epithelial phenotype. The decline in ESRP1 expression coincides with the switch in expression from CD44v to CD44s. ESRP1 negatively regulates EMT by preventing CD44 isoforms switch in breast cancer cells [54]. Knocking down ESRP1 in CD44v-expressing cells resulted in an isoform switch to CD44s, leading to suppression of lung colonization [70]. A study of 123 PDAC patients indicate that overall survival rate of the ESRP1-high group was significantly longer than that of the ESRP1-low group [153]. Our study show that epithelial like PDAC cells express high level of ESRP1 but mesenchymal like PDAC cells have little to none ESRP1 as detected by RT-PCR and Western blotting [12]. The expression of Snail and Zeb1 function as transcriptional repressors of ESRP1 and induces EMT [154, 155]. Snail-mediated ESRP1 repression results in a decrease in the levels of CD44v and an increase in production of the CD44s isoform, which drives EMT in breast cancer cells. Ectopic expression of ESRP1 inhibits Snail-induced EMT and that re-expression of CD44s isoform rescues the impaired mesenchymal phenotype. CD44 is a major splicing target downstream of ESRP1 during Snail-induced EMT [155].

We were recently able to separate cancer cells on the bases of CD44 expression level using flow cytometry [12]. This study showed that cells possessing an EMT phenotype expressed high levels of CD44s with lower levels of CD44 single exon variants. Mice with orthotopic implants of CD44s high cells formed tumors more rapidly and gradually developed resistance to gemcitabine, whereas tumors from CD44 low expressing cells took longer to grow and maintained sensitivity to gemcitabine over an extended period of time. However, tumors originated from CD44 high/EMT/chemoresistant cells showed expression of CD44v suggesting a dynamic switch of CD44 isoforms during tumor formation. Thus, CD44s may play a role in EMT and tumor spread and CD44v may play a role in tumor engraftment and growth [12].

### CD44 and chemoresistance

#### CD44 and drug resistance of the cancer cells

The emergence of chemoresistant cancer cells may result from the expansion of a subpopulation of cancer cells that are inherently resistant or by reprograming or phenotypic changes of initially sensitive cancer cells induced by the stress of chemotherapy. We found that treating a pancreatic cancer cell line BxPC-3 with gemcitabine induces cells to undergo an EMT, to become more invasive, and to show a switch from expressing mainly low levels of CD44v to expressing high levels of CD44s [156]. This mesenchymal/CD44s phenotype was reversed after multiple passages following removal of gemcitabine. Chemotherapy or other environmental stresses may induce a CD44s high expressing phenotype that promotes survival and that are more invasive. Inhibiting or reversing the switch toward a mesenchymal phenotype may prove to be an important strategy for increasing the response to chemotherapy.

Clinical evidence showed a positive correlation between CD44 expression and breast cancer bone metastasis [157]. In vitro, knock-down of CD44s expression in breast cancer cell lines leads to markedly decreased number of tumor sphere formation in suspension cultures. Cell migration and invasion were suppressed by CD44s knock-down. Overexpression of CD44s increased tumor sphere formation and cell migration. In vivo, tumor formation was inhibited in mice orthotopically inoculated with CD44s knock-down cells. The number of osteoclasts decreased in the bone metastases of CD44s knock-down tumors [158]. These studies suggest cancer cells gaining acquired drug resistance tend to be accompanied with relatively higher expression of CD44s isoform.

Chemoresistance can be mediated through expression of P-glycoprotein (MDR1) coded by the multidrug resistance (MDR) gene. P-glycoprotein either reduces drug uptake or causes efflux of the drug out of cancer cells. MDR1 has also been linked to an increase in hyaluronan production in drug-sensitive tumor cells [159]. This study indicated that HA and CD44 binding constitutively activates phosphoinositide 3-kinase (PI3K)/AKT signaling, which enhanced P-glycoprotein activity in doxorubicin-resistant human breast carcinoma cells. Another study suggested HA, PI3K, and ErbB2 form a positive feedback loop to amplify MDR1 expression. HA and CD44 interaction activated an ErbB2-containing signaling complex, stimulating PI3K activity in multidrug-resistant human breast carcinoma cells. PI3K-activated AKT and downstream anti-apoptotic pathways contributing to drug resistance [160]. Transient knock-down CD44 from placenta-derived human mesenchymal stem cells suppressed the hyaluronan-substratum-induced drug resistance and resulted in fewer MDR1-positive cells [161]. Gemcitabine-resistant pancreatic cancer cells HPAC and CFPAC-1 were treated with serially escalated doses of gemcitabine. The resistant cells form greater numbers of colonies; they were also able to form more tumor spheres in serum-free medium than their counterpart parental cells. Those gemcitabine-resistant cells were more tumorigenic in vivo confirmed by subcutaneous xenografts. FACS analysis had shown that most of the repopulated cells after surviving high-dose gemcitabine were CD44 positive. These studies suggested that CD44 plays a role in chemoresistance although the mechanisms are still under the investigation [148].

#### CD44 and apoptosis of the cancer cells

CD44 plays an indispensable role in activating survival pathways that protect cancer cells from apoptosis. The role of CD44 in cell survival involves alteration of multiple molecules that regulate pre or anti-apoptotic processes. These included expression of Fas, caspase 3/9, Bcl-xl/Bak, phosphorylation of AKT, pRb, and upregulation of anti-apoptotic Bcl-2 [162–164]. CD44s, CD44v3-v10, and CD44v8-v10 transfected human colon cancer cell line promoted resistance to etoposide-induced apoptosis. Bcl-2and caspase-3 expression was elevated in both CD44s- and CD44v-transfected cells [162]. Knock-down of CD44 from colon cancer cells lead to reduced expression of anti-apoptotic molecules like Bcl-2, Bcl-xL and increased level of apoptotic molecules like Bax, caspase-3/8/9 [164]. AKT phosphorylation, p21, and pRb were downregulated in CD44-transfected cancer cells after anticancer reagent etoposide treatment. This suggests that expression of CD44 modulates cell cycle regulators pRb and p21, and the pro-survival protein AKT [162]. CD44 also contributes to cell survival via regulating Fas in lung cancer cells. HA binding to CD44 downregulated Fas expression and reduced Fas-mediated apoptosis [163].

CD44 expression was found enriched in CLL patients. Ligands-stimulated CD44 promoted CLL cells survival. siRNA-mediated CD44 knock-down CLL displayed dimished cell viability, even in AKT-overexpressing CLL cells, suggesting that CD44 was a key survival mediator. Targeted deletion of CD44 in murine leukemogenesis model decreased the tumor burden in peripheral blood and spleen, resulting in a prolonged overall survival. MCL1 level, anti-apoptotic downstream effector of CD44 in CLL, was remarkably reduced in CD44-deficient mice. CD44 regulated anti-apoptotic MCL1 expression via the ERK and AKT pathways [45].

HA binding to CD44-induced tyrosine phosphorylation and activation of FAK which associated with PI3K. CD44 expressing cells were significantly resistant to etoposide-induced apoptosis. Inhibition of FAK or PI3K canceled out CD44 anti-apoptotic effect [165]. HA-CD44 interaction activated ErbB2-containing signaling cascade including Hsp90, cdc37, p110, and p85 [166]. Therefore, CD44 may provide a protection mechanism for cancer cells from apoptosis.

## Therapeutic strategies for targeting CD44

Targeted therapies are designed to block aberrantly activated signaling pathways in tumor cells specifically and are often tolerated better than conventional chemotherapies. CD44 may serve as a therapeutic target due to its role in regulating multiple survival signaling pathways and overexpressing CD44 is considered as a CSC marker. The major categories of CD44-targeted therapies include neutralizing antibodies, peptide mimetics, aptamers, pharmacological inhibitors, CD44 decoys, and HA oligomers. Some of the current approaches used to target CD44 are discussed below.

### Antibodies to CD44

Antibodies to CD44 are being studied in preclinical and clinical trials for cancer therapy. CD44 antibodies conjugated to anti-tumor agents can be used to target CD44-expressing cancer cells or netballing antibodies that block CD44-mediated signaling were being developed for cancer therapy.

For example, humanized monoclonal antibody (mAb) Bivatuzumab (BIWA-4) combined with microtubule inhibitor mertansine and BIW1, derived from VFF18 conjugated to an antimicrotubule agent to target CD44v6 in head and neck squamous cell carcinoma. However, these clinical trials were discontinued because of severe skin toxicities with one fatal outcome attributed to mertansine conjugates [167, 168]. Another mAb U36 was characterized by cDNA cloning in COS-7 cells. Peptide sequencing and screening data revealed that U36 antigen was identical to the squamous cell specific CD44v6 [169]. The mAb U36 was labeled with indium-111 and intravenously injected to nude mice bearing HNSCC xenografts expressing CD44v6. The mAb U36 had significantly higher uptake in tumors [170]. Moreover, the murine monoclonal IgG1 antibody VFF18, specific for human CD44v6, showed fast and selective tumor uptake of the radioimmunoconjugate in nude mice bearing human epidermoid carcinoma xenograft [171].

In addition, a humanized mAb specific for CD44 (RG7356) was found to be cytotoxic to leukemia B cells without affecting the viability of normal B cells in chronic lymphocytic leukemia cells. Interestingly, the effects of RG7356 were not neutralized in the presence of HA [172]. In contrast, anti-CD44 blocking antibodies (IM7 or KM201) inhibited HA-induced VEGF generation in human vascular endothelial cells [16]. Invasive potential of breast cancer cells with a mesenchymal phenotype can be inhibited by rat anti-human CD44-specific antibodies (IM7) [173]. Treatment of mice harboring orthotopically implanted pancreas tumors with a CD44 neutralizing antibody (H4C4) reduced tumor growth, metastasis, and postradiation recurrence [13].

Roche has developed a novel functional mAb (RO5429083) which targets a glycosylated, conformation-dependent epitope of CD44. Based on preclinical investigations, RO5429083 and its radioimmunoconjugates were approved and used in several clinical trials. This study was conducted from 2011 to 2014 to assess the pharmacokinetics, pharmacodynamics, safety, and efficacy of RO5429083 in patients with metastatic and/or locally advanced CD44-expressing malignant solid tumors. First cohorts of patients received RO5429083 intravenously at escalating doses. Second cohorts of patients received 89Zr-labeled RO5429083 in cycles 1 and/or 2, followed by RO5429083. All patients had an option to continue treatment with RO5429083 until disease progression or unacceptable toxicity occurs (NCT01358903, NCT01641250, NCT01358903; ClinicalTrials.gov). In 2016, a dose-escalation clinical trial study in patients with advanced gastric cancer was conducted. CD44 variant was shown to interact with xCT, a subunit of cystine-glutamate transporter, which maintains high levels of intracellular-reduced glutathione (GSH) which defend the cell against oxidative stress. Sulfasalazine, an inhibitor of xCT, was shown to suppress the proliferation of CD44v-positive cancer cells both in vitro and in vivo [174]. Another ongoing clinical trial study (ClinicalTrials.gov NCT01577511) was related to invasiveness and chemoresistance of CSC in colon cancer. Circulating tumor cells (CTCs) potentially have CSC phenotype and CD44 is considered as its biomarker. CD44v6 expression was strongly enriched in CTC lines; immunostaining for CD44v6 and the CSC marker ALDH was strong in tumors grown after subcutaneous CTC injection in immunocompromised mice [175]. Those studies support the continued development of therapeutic strategies to target CD44 expression in patients.

### Peptides and aptamers

Other than antibodies, another strategy is to utilize the synthetic peptides that block CD44-HA interactions or to reduce CD44 expression in cancer cells. Mice treated with PEP-1 a CD44 binding peptide showed a lower gastric stem cell proliferation. Treating CD44 double knockout mice with PEP-1 did not further reduce the rate of proliferation suggesting that CD44 may play an indispensable role in maintaining normal stem cell turnover. PEP-1 can reduce CD44 expression in gastric cancer cells [146]. A novel CD44-binding peptide from the pro-matrix metalloproteinase-9 hemopexin domain impairs adhesion and migration of CLL cells [176].

Aptamers have high binding affinity and specificity for target proteins to inhibit their functions. Peptide, DNA, or RNA small synthetic aptamers have been developed [17]. CD44v10 proteins formed complexes with the surface protein EphA2, which contributes to the invasiveness of cancer cells. DNA aptamers that specifically bound to CD44 exon v10 inhibited the migration of breast cancer cells [18]. 2′-F-pyrimidine-containing RNA aptamer (Apt1), selected against CD44, was successfully conjugated to the surface of PEGylated liposomes. The cellular uptake for Apt1-Liposome (Lip) was tested among two CD44 (+) cell lines, human lung cancer cells (A549), and human breast cancer cells (MDA-MB-231) compared to CD44 (−) cell line, mouse embryonic fibroblast cells (NIH/3T3). A higher sensitivity and selectivity in CD44(+) cancer cells was observed for Apt1-Lip compared to the blank liposomes [177]. An RNA-based bispecific CD44-EpCAM aptamer was developed and tested in preclinical models. It can block CD44 and EpCAM simultaneously by fusing single CD44 and EpCAM aptamers with a double-stranded RNA adaptor. The bispecific CD44-EpCAM aptamer was significantly more effective than either single CD44 or EpCAM aptamer for inhibiting the cells growth or inducing apoptosis in ovarian cancer cells both in vitro and in vivo studies [178].

### Pharmacological inhibition of CD44

In addition to directly targeting CD44, several natural compounds or chemotherapeutic drugs were shown to indirectly inhibit CSC which express CD44 isoforms [179]. Silibinin, a major bioactive component of the plant *Silybum marianum*, has been studied extensively for its efficacy in prostate cancer and in phase II clinical trials. Silibinin inhibited CD44 promoter activity, caused a 90% inhibition of total CD44, and decreased the levels of CD44v7-10 RNA and protein in prostate cancer cells. Silibinin also caused a dose- and time-dependent cell growth inhibition in pancreatic cancer cell lines (BxPC3 and Panc1) both in vitro and in vivo [180–182]. Zerumbone, a monocyclic sesquiterpene derived from a Southeast Asian ginger, is often used as an anti-inflammatory and antioxidant agent. Zerumbone downregulated the basal level of CD44 expression in breast cancer cells. Zerumbone suppressed EGF-induced CD44 expression by inhibiting phosphorylation of STAT3 [183–185]. Gemini, a family of vitamin D analogs, is active in gene transcription with enhanced anti-tumor activity. Gemini vitamin D decreased CD44 protein level, inhibited the invasion of the basal-like human breast cancer cells, and reduced tumor size in an orthotopic mice model [143, 186]. Combined treatment of breast cancer cells with curcumin and epigallocatechin gallate (EGCG) reduced CD44-positive CSC population and decreased phosphorylated STAT3 expression. This suggested these two phytochemicals may inhibit CD44 expression [145]. The flavonoid apigenin also inhibited CD44-positive CSC and prostate cancer cell survival in a dose-dependent manner and the mechanism might be due to a significant increase in expression of p21 and p27 [187].

### HA-directed targeting of cancer cells

To maximize the drug effect on CD44 overexpressing cells, CD44 major ligand HA was used as a carrier to deliver chemotherapy agents. HA-drug bioconjugates, such as HA-Taxol, HA-CPT11, HA-Paclitaxel (PTX), and HA-polymer conjugates, can efficiently bind to CD44 overexpressing cancer cells and exert strong anti-proliferative activity on CD44 overexpressing cancer cells including human breast, colon, ovarian, gastric, breast, esophageal, and lung cancer cells as well as rat colon adenocarcinoma and colorectal cancer cells [188–190]. Paclitaxel (PTX) conjugated to these HA-polymers display better cell killing effect on cancer cells than treating them with PTX alone. Of note, the lower molecular weight oligomers of HA had better drug delivery effect by targeting CD44 overexpressing ovarian cancer cells [190].

CD44 possessing cells have been targeted by HA-containing nanoparticles. Mice inoculated with hepatocellular carcinoma cells were orally administered with HA-selenium nanoparticles at different doses. The HA-selenium nanoparticles showed less toxicity and significantly reduced tumor weights compared to a 5-fluorouracil positive group [191]. Similarly, the efficacy of doxorubicin loaded hyaluronan-coated superparamagnetic iron oxide nanoparticles has been evaluated in subcutaneous nude mice which intraperitoneal injection of human SKOV-3 ovarian cancer cells. HA-doxorubicin nanoparticles distributed wider in the tumor tissue than intravenously injected free doxorubicin, leading to significant reduction of tumor growth and increased the lifespan of those mice [192]. HA-encapsulated siRNA nanoparticles have been investigated. A biodegradable nanoparticle improved the delivery of CD44 and FAK siRNAs to ovarian cancer xenografts and reduces the tumor growth [193]. HA-encapsulated epigallocatechin-3-gallate were efficiently internalized into cancer cells via CD44 ligand receptor recognition, induced cell cycle arrest at G2/M phase, and inhibited prostate cancer cell growth. In vivo studies showed that these nanoparticles specifically bound CD44 receptors and increased apoptosis of cancer cells, leading to significant decreases in prostate tumor growth [194].

Chemotherapeutic agent-encapsulated liposomes, or covalently bound HA-bioconjugates, were selectively taken up by tumors in systemic delivery, and reduced specific gene expression in several xenograft models [195]. For example, PTX mixed with lipid molecules and assembled into lipid clusters and subsequently covalently coated with HA to form HA-Lipid-PTX nanoparticles have been examined in preclinical models. CD44-positive breast tumor cells (MDA-MB-231) exhibited more efficient internalization of the HA-coated nanoparticles compared with CD44-null normal cells (NIH3T3, CV-1). MDA-MB-231 cells appeared more sensitive to the cytotoxic activity of HA-coated nanoparticles. In vivo, the tumors from mice in the HA-Lipid-PTX treatment group were significantly smaller compared with the control group [196].

Nano-sized self-assemblies based on amphiphilic iodinated HA were developed for use in cancer diagnosis and therapy. 2, 3, 5-Triiodobenzoic acid (HA-TIBA) was conjugated to an HA oligomer as a computed tomography imaging modality and a hydrophobic residue. Nanoassembly based on HA-TIBA was fabricated for tumor-targeted delivery of doxorubicin (DOX). Cellular uptake of DOX from nanoassembly, compared to a DOX solution group, was enhanced via an HA-CD44 receptor interaction [197].

### CD44 decoys and HA oligomers that inhibit CD44-HA interaction

Several strategies have been used to block HA-CD44 binding. The soluble CD44 ectodomain was used as a competitor of CD44 on tumor cells to compete out HA ligand as a potential therapeutic approach to inhibit tumor growth and metastasis [198]. Soluble CD44s almost completely inhibited HA binding on melanoma cells, whereas soluble CD44 mutated in the HA binding domain had no effect. Human melanoma cells transfected with an expression construct for the CD44 ectodomain showed retarded tumor growth [198]. Several fusion proteins composed of the constant region of human IgG1 fused to the extracellular domain of human CD44v3-v10, CD44v8-v10, or CD44s have been developed. Expression of these fusion proteins reduced binding of HA to glioblastoma cells. Those fusion proteins markedly inhibit subcutaneous and intracranial cell growth and significantly extended the survival of mice-bearing intracranial tumors [133].

The disruption of HA-CD44 interaction by using HA oligomers was another approach to target CD44. This approach involves replacing the multivalent interaction of high molecular weight (HMW) HA and CD44 with monovalent interaction of small oligomers of HA (6–18 saccharide units of HA). For example, HMW HA was found to augment chemokine CXCL12-induced CXCR4 signaling in both HepG2iso cells and primary human umbilical vein endothelial cells, accompanied with enhanced ERK phosphorylation and increased cell motility. The augmentation of CXCR4 contributed to increased vessel sprouting and angiogenesis. Small HA oligosaccharides (sHA) efficiently inhibited these effects CD44 and CXCR4 were found to physically interact in the presence of CXCL12, an interaction that could be inhibited by soluble HA [199].

## Conclusion

CD44 is upregulated in a variety of cancers and can be expressed in its standard isoform, CD44s, or as a number of alternatively spliced variant isoforms, CD44v. CD44 isoforms are overexpressed in many cancer types. Here, we discussed the current understanding of the structure and function role of CD44 in the pathogenesis of cancer.

CD44 mediates its effects on the cancer cell by activating signaling pathways including protein kinases, by activating transcription factors and by modulating the cytoskeletal architecture. The functional role of CD44 is pleiotropic, includes inducing EMT, altering the cellular cytoskeleton, by promoting drug resistance and through anti-apoptosis mechanisms. Proteomic and genomic approaches can identify the mechanisms by which CD44 promotes tumorigenicity. CD44 can act as co-receptors and/or stabilize receptor tyrosine kinase receptors causing tumor cell proliferation and invasion. CD44 cytoplasmic tail often binds to adaptor protein to initiate cytoskeletal changes and cell signaling pathways including MAPK, Hippo, β-catenin, AKT, TGF-β, Emmprin, MMPs, and STAT3. Expression of the CD44s isoform appears to contribute to EMT and tumor invasion. Multiple CD44v tends to play a role in epithelial phenotype, tumor initiation, growth, and are often associated with metastatic lesions. The functional roles of various CD44 isoforms on cancer development and progression remain an active area of investigation.

CD44 expression is regulated by multiple levels including transcription factors, epigenetic mechanism, microRNAs, and by post-translational modifications. ESRP1 is essential for promoting exon inclusion of CD44 isoforms and is required for expression of CD44v isoforms. The downregulation of ESRP1 accompanied with a switch from CD44 variants to CD44s isoform is associated with EMT. The switch of CD44 isoforms as how this relates to their functional roles in adaptive plasticity of tumor cells is becoming more clearly delineated.

A number of studies validate the potential of CD44 as a therapeutic target in various tumor types. Current therapeutic strategies include neutralizing antibody, peptide mimetics, aptamers, natural compounds that suppress CD44 expression, HA-directed targeting of bioconjugates and nanoparticles, HA oligomers, and CD44 decoys. These studies are in various stages of preclinical and clinical trials.

## Abbreviations

ALDH1: Aldehyde dehydrogenase-1
BPH: Benign prostate hyperplasia
CAF: Cancer-associated fibroblasts
CD44s: CD44 standard
CD44v: CD44 variants
CLL: Chronic lymphocytic leukemia
c-MET: c-MET receptor tyrosine kinase
CRC: Colorectal cancer
CS: Chondroitin sulfate
CSC: Cancer stem cell(s)
CTC: Circulating tumor cells
DOX: Doxorubicin
EDA: Fibronectin extra domain A
EMT: Epithelial to mesenchymal transition
ERM: Ezrin-Radixin-Moesin
ESRP: Epithelial splicing regulatory protein
FAK: Focal adhesion kinase
HA: Hyaluronic acid
HAS1: Hyaluronic synthases1
HGPIN: High-grade prostatic intraepithelial neoplasia
HNSCC: Head and neck squamous cell carcinoma
MAMCs: Malignant-appearing microcalcifications
MD: Mammographic density
MDR: Multidrug resistance
MET: Mesenchymal to epithelial transition
MMPs: Matrix metalloproteinases
OPN: Osteopontin
PCa: Prostate cancer
PDAC: Pancreatic ductal adenocarcinoma
PDGF: Platelet-derived growth factor
PTX: Paclitaxel
RHAMM: Hyaluronic acid-mediated motility receptor
SCID: Severe combined immune deficiency
SDF-1: Stromal-derived factor 1α
VCAN: Versican
VEGF: Vascular endothelial growth factor
